# The Etiology and Treatment of Inebriety

**Published:** 1881-09

**Authors:** Charles Warrington Earle

**Affiliations:** Physician to the Washingtonian Home, and Prof. Diseases of Childred, Women’s Medical College, Chicago


### ARTICLE III.

**THE ETIOLOGY AND TREATMENT OF INEBRIETY.** By Dr. CHAS. WARRINGTON EARLE, Physician to the Washingtonian Home, and Prof. Diseases of Childred, Women's Medical College, Chicago.

During the past ten years the writer has had unusual opportunities for investigating the causes of inebriety, as assigned by the men themselves. The results of treatment in the Washingtonian Home of this city have been noted, and the deficiencies, especially the want of a proper place for what I denominate the "third class," have been fully appreciated. Some of these reflections and conclusions, it appears to the writer, may be of some value to the medical profession.

In the first place, then, why do men commence the use of stimulants ?

*Second.* After periods of reformation why do men again commence the use of alcoholics ? and,

*Third.* How shall we treat them ?

We are in medicine constantly speaking of the cause of certain phenomena, and, having found it, it comes to be an everyday expression in the treatment of patients, " Do away with the cause ;" and so at the outset comes the question, " Why do men commence to drink stimulants ?"

Answers based upon actual questions to many hundreds, and extensive observation, authorize me in saying, in regard to the

FIRST CAUSES OF DRINKING,

*that association with drinking companions* stands at the head of the list—forty per cent. giving this as the primary cause of deviation from a temperate life. In the majority of these cases the habit commenced in youth, and the particular stimulant used was *beer*, or whisky considerably diluted and flavored. It is the mild alcoholic at first, generally changed to the stronger as the appetite becomes accustomed and demands them.

*Sociability* comes next on the list, *ten* per cent. giving this as the reason. The custom of " treating," as it is called, is regarded as an evidence of sociability, and, in certain classes, is carried to a great extent. The first in a group of friends orders a particular alcoholic, and it is not strict sociability until all have imitated the example.

*Trouble of various kinds*, either in business or in the family, is assigned in ten per cent. of the cases. It will be observed, however, that after the habit is formed trouble of any character will, in a *greater* number of cases, be given as a cause of relapses.

*The custom of drinking in families*, so common a half century ago, seems to influence but few, and it is hoped that the time is not far distant when it will cease to be a cause. *Two per cent. only* acquire this habit in their homes. As a first cause this habit cannot be too strongly condemned, and the people need education particularly on this subject. I am inclined to think that a much larger number owe the formation of an appetite to this cause than my figures seem to show. I frequently have to expostulate and condemn the practice, especially in those families where beer or wine is used to some extent. A few drops are given to the children. This I have noticed in some of the best foreign families whom I attend, and that it is regarded in the least dangerous to the future welfare of their children is quite a matter of surprise to the parents. As I remarked before, they need education in regard to it. Let them know the facts, and they will stop it.

*Different kinds of business*, which bring a person in contact with various kinds of alcoholics, has been a temptation but few can stand, and those engaged in the liquor traffic and in the hotel business sooner or later contract the habit, although a few, beginning to feel its effect in their own bodies, or noticing it in others, stop, and are temperate men.

*Mental depression* and active brain work are not frequent causes until after the habit is once formed.

Many young men, and a few of riper years, date the commencement of drinking habits to *army and navy associations*. During the war of the Rebellion many of the present generation were called from the restraining influences of home to share the vicissitudes of camp life and the excitement of the battle-field. It was at a time when they were forming habits for good or evil, and it can hardly be regarded with surprise that some contracted the habit of using alcoholic stimulants, which has followed them to this day. About *ten per cent.* give this as the first cause of their drinking. The habit is contracted at college by a few, and a great variety of excuses are given by others as the first cause of their drinking. A natural love for liquor is claimed by a few—exhaustion, poor health, sickness, etc., etc., are also cited as reasons.

I am thoroughly convinced that the great majority of boys and young men commence the use of alcoholics without the least idea that it is a dangerous practice. At first it is social; the preparations are agreeable to the taste, and the effect at this time, from the fact that only a small amount is taken, is not perceptible. What the youth of our country need is education as regards the properties, and particularly the effects, of all kinds of alcoholics on the human system. It should be understood by every youth that the moderate use of mild alcoholics is not only exceedingly dangerous, but in a large number of cases, from causes from which I will presently enumerate, is absolutely ruinous. They should be taught to handle it as they handle aconite or arsenic.

I need not say here, for you will notice all through my paper that I believe that our only hope is in the education of the young. The great mass of those addicted to the intemperate use of liquor to-day will never be temperate citizens. Many of them will have spasmodic periods of teetotalism, only to succumb to the appetite, and for a time lapse into their former habits.

This brings me then, to speak of

RELAPSES, or to consider the "Second Part" of my subject.

After periods of reformation, why do men relapse into former habits?

Unfortunately many who have apparently commenced a successful reformation begin to drink again. This unfortunate circumstance on the part of men who have stood up well for years causes, I think, more anxiety and discouragement than anything else connected with the treatment of inebriates.

It is not a difficult thing, after a period of intoxication, with the disgrace and physical infirmities conspicuously present to one's own self to induce the victim of this unfortunate habit to abstain from further drinking for perhaps several months, but many, as I remark above, again drink. To these men the question propounded has been, "What reason do you assign for drinking since you attempted reformation?" In the main I believe, as I do in regard to the reply given to my first question, that the answers are conscientiously and truthfully given. In the majority of cases it is a subject which has been seriously and carefully contemplated. The young man who is forming the habit of drinking, surrounded by gay companions, and taking the mild alcoholics, does not give the subject of reformation a thought; but when a person begins to feel the power of the stimulant over him, and especially after he has lost his business and ruined his family, he reviews with great earnestness the causes of his relapse.

At the head of the list must be placed *trouble.* After the man has once formed the habit, the moment trouble comes, either in his family or in his business, or from grief at the loss of friends, or in disappointment at the loss of position, the unhappy man rushes for some variety of alcoholic stimulant. The man does this, too, fully realizing what it will do for him. It is the sheerest nonsense to say that he cannot help it. He does not want to help it. What he *does* want is to become oblivious to his trouble, and he knows that alcohol will produce this desired condition. Alcohol is one of the anæsthetics, and under its influence trouble, and sorrow, and disappointment are forgotten. *Sixty per cent.* of our relapses come fairly under this category.

*Dissolute and wicked companions*, either male or female, rank second in the causes of relapse. The young man just contracting the habit does not usually associate with men who are absolutely stained with crime, and he may continue for years to occupy his position in good society, and may enjoy to a considerable degree the confidencc of his friends. Sooner or later, however, he commences to fall into bad company, and by the time his attention has been directed to any reformatory measures he has been discarded by many of his friends, and he has become the associate of the dissolute and outcast of the earth. A man of this character, who from any cause has had his attention directed toward reformation, always finds it a hard task to accomplish. Everybody, except his evil companions, has lost all confidence in him, and however well he may do, it is with great difficulty that his friends are induced to assist him in the least. They have long since ceased to give him more than a passing thought, and he is ostracised by all save his boon companions. It seems cruel that a man, under almost any circumstances, can procure a drink from a saloon-keeper, while the same individual, when trying to gain a foot-hold after the commencement of a reformation, *cannot get a cup of coffee*, and *hardly* the crumbs which fall from the rich man's table. I have frequently seen a man completely discouraged by the refusal of his friends to help him to merely a suit of clothes, or to secure a subordinate position. Rejected by his friends, disappointed in every effort to obtain an honest livelihood, he knows of one class of people who will share all with him, and in his desperation he goes back to his old companions. *Twenty-five per cent.* relapse in this manner.

*Bodily suffering and chronic diseases.* Many persons never enjoy perfect health. They suffer from a feeling of prostration. They are not robust and healthy. This may come from their parentage, or from subsequent illness. These people may have acquired the habit from some of the first causes I have enumerated. They have learned from experience that stimulants, for a short time at least seem to supply this want of nerve energy. It will bridge over, as it were, the feeling of prostration. The time comes, however, when this class of people desire to stop the use of alcoholics. They place themselves in an institution where, surrounded by temperance influences and supported by good diet and tonics, their reformation seems quite probable. An exposure, however, to causes which develop their physical weakness, produces in a number of cases a relapse. Men too, who are suffering from exhaustion and various painful diseases, have repeatedly told me that it was the cause of their relapse. A gentleman suffering from lupus of the face, of twenty years standing, assured me that it was the cause of his continued drinking. Patients who suffer from chronic lung disease frequently take alcoholics to quiet pain and relieve exhaustion.

*At least fifteen per cent.* fall from the causes just enumerated.

*Night work*, and the occupation known as "commercial traveling," cause many men to fall who had made every promise of success. Several years ago I made an estimate based on the admission of a thousand men into our institution who worked nights, and whose occupation deprived them of home influences and exposed them to an irregular and changeable method of living. *Fourteen per cent.* of the number I made the calculations upon, had relapsed from this cause. Recently I have carefully verified these figures, and find that almost without an exception the habit was formed before these men engaged in business, and this mode of life caused a *relapse.*

*The absolute love* for alcoholics, both as regards its taste and its pleasant effects when indulged in moderately, is a cause of a few relapses; but there is found in a majority of these cases some other facts, such as trouble, reverses, or evil companions. Certain customs, which are regarded by some of our most radical reformers as prolific causes of intemperance, I find exercise a very insignificant place among the reasons assigned for drinking at first, or as a cause of relapses. I allude to the prescribing of stimulants in combination with certain medicines by physicians; to the habit of offering wine to visitors on New Year's, and the partaking of wine at the communion-table. I have found two men who claim the first as the cause of relapse—one from taking wine on New Year's day, and have heard of those, but have never seen one, who dated his downfall from partaking of the sacrament.

I do not desire to have it understood that I favor the first custom, only when absolutely necessary, or that I advocate the second, somewhat ancient and fashionable habit of society, or commit myself as regards the propriety or impropriety of the use of ordinary wine in churches. My deductions are statistical. I can say this, however, that notwithstanding all that has been said and written against the use of alcohol as a medicine used properly and when needed to save life, there is no considerable number of physicians of any sect or school who feel that their entire duty is performed in the treatment of prostrating and septic diseases until the properties of alcohol as a stimulant and disinfectant have been tried. I was educated in a hospital where alcoholics were sparingly, almost beggarly, used, and I have stood by the bedside of the man with pneumonia and seen him die, whereas now I know, with ten years' observation, that a proper amount of this drug would, in all probability, have saved him. I have listened to the windy and stentorian arguments of the temperance lecturer and heard him declare that he would prefer—aye, a hundred times—prefer death to the prescribing of any alcoholics to him, even if on his death-bed. I have seen the same man and men on their sick-bed, not dangerously ill, but comparatively so, and long before there was any demand for it, have been told to exercise my own judgment in regard to the matter of stimulation.

In regard to communion wine being a cause of intemperance, I desire to say that particular stress and importance is paid to this by some at least who have no sympathy whatever with the church. If a single person has made the statement that he was tempted by partaking the elements commemorating the death and crucifixion of our Saviour, the statement has been exaggerated and frequently misrepresented by men guilty of profanity and licentiousness, and who never for a moment stop to consider how many hundreds have been saved by the influence of these organizations.

This may be somewhat foreign to my subject; but presently in this paper will come the question, "Who is to wage the fight against intemperance?" Is the attacking party to be made up of a few temperance societies, who are constantly berating the churches for fostering and aiding, and tempting men to become drunkards? Not by any means. Every temperance man and woman, whether they can subscribe to all the ultra ideas of the most stalwart, should be enlisted in the work, and the most ultra should receive them, and then, on a platform judiciously formed, wage a warfare, not in a way that invites disaster, but in a manner which will enlist the aid and support of the masses.

### SHOULD IT BE TREATED AS A DISEASE?

A theory is being constantly urged, particularly by *Superintendents and Physicians of Inebriate Asylums*, that "*Drunkenness*" is a *disease*—not a *vice*. Their ideas and peculiar views find ready believers and advocates in the friends of the more aristocratic class of those addicted to the intemperate use of whiskey, and among the victims themselves who do not *care to attempt* reformation. I am not aware that anybody ever thought drunkenness in *a poor man a disease*—it is always a class that are able to pay large fees to specialists, and those able to board at asylums at $50 per week (more particularly in the East) and coddled with the idea that they have a peculiar pathological condition, that we find the largest number of advocates of this (to me) pernicious and destructive theory.

As early as 1872, I placed on record in an article published in the CHICAGO MEDICAL EXAMINER, certain observations in regard to the newly discovered disease, inebriety. They were directly and explicitly opposed to the theory, and after nearly ten years' additional experience, during which more than 2,500 cases of alcoholism and 1,500 complications have been treated, my convictions remain unchanged. It is probably just for me to say, however, that a large number of physicians, particularly those engaged in the specialty of nervous diseases, believe alcoholism to be a disease.

I desire at this time to again affirm—

1st. That in my opinion there exists no physical cause which compels a man to drink intoxicating liquors.

2nd. That 99 out of every 100 men who have formed the habit *can* reform if they desire; the remaining one per cent. are probably insane and should not be held accountable. The trouble is, men will not use the means for reformation which are freely and entirely at their command. They desire to reform, they say, and yet insist in indulging in all other previous bad habits and associations. They say they desire to reform but refuse any cultivation of the moral sense, any strengthening of the will. They desire a reformation which costs but little or no effort—a *cure* which will enable them to practice all previous bad habits, mingle with all previous vicious associates, gratify all the lower feelings and passions, and to all of which I might add, use alcoholics with moderation, with no danger of ever becoming intoxicated. This is the kind of cure many desire. They do not want reformation.

I am constantly having gentlemen under my observation, formerly addicted to the use of alcohol, men of great business capacity, men of fine moral sense, and with wills powerful enough to carry them directly in the way of duty, notwithstanding any reverses, disappointments or temptations, and yet they believe in the so-called disease inebriety, and that they are victims of the malady. With these splendid business qualifications, with keen moral and religious senses, and with will powers equal to any emergencies, they clamor for some *specific medication*, which will do away with the appetite, which will enable them to take a single drink without the desire to take another. A man is reformed when his moral sense teaches him that it is wrong, and his will power is sufficiently strong to enable him to abstain from taking the first drink, and for such a person to assert that he is the victim of a disease is to me nonsense. It should be remembered that we are now speaking of men who have acquired the taste for stimulants, and have a desire for them. We speak of the desire or motive for taking them as the disease, not the condition they are in after the stimulant has been taken into the system.

Dr. Parrish, in the Third Annual Report of the American Association for the Cure of Inebriates, speaking of consumption and alcoholism says: "The cough, hectic flush, night sweats and wasting of tissue are no more symptoms of disease in the one case than the injected and glaring eye, flushed face, confused speech and staggering gait in the other."

To me there is no comparison whatever between the two cases. Place these two patients in a room; supply the consumptive with a good generous diet and what medicine is necessary; supply the patient with the so-called alcoholic disease with a little nerve sedative, some beef tea, etc; visit them at the end of four days, and mark the progress of their respective diseases. The consumptive has continued to fail; his emaciation is greater; his strength is not so good; he is worse. How is it with the other? His injected and glaring eye has disappeared; his face is no longer flushed; his speech is intelligible; his gait steady. In a few days he is ready for business. He will now stop drinking if he earnestly desires. In order to accomplish this, however, he must do what every man must do who is peculiarly tempted by appetite or passion—carefully avoid the temptation, and in every possible way earnestly and zealously perfect a noble manhood, and reformation will certainly crown the effort.

Alcoholics produce very grave changes in almost every tissue of the body, and as I have said, and shall say again, produce disease of every variety. But it is the motive, or impulse, or desire that is called the disease. If a person is not responsible for an evil motive, or impulse, or desire, then we should not hold him responsible for all the calamities which come from partaking of alcohol. A man is not responsible for acts which absolute structural changes in his body compel him to do. I grant this of course; no one pretends that a man is to blame for the palpitation of a diseased heart induced by a rheumatism, or for an enlarged spleen caused by chronic malarial poisoning. It is beyond the power of the will to prevent these maladies; they are of the body; they are diseases; we cannot avoid them; they have a previous history and a regular course. There is a cause for the structural changes which really exist.

And now about Inebriety: Is there any previous history? Any cause except the voluntary taking into the system a certain amount of liquor? Has any structural change taken place in the persons of the hundreds when the first drink is taken? Is there any inherent propelling cause why our pastors or our deacons should go out and take enough liquor to render them absolutely unconscious; and yet pastors and deacons, and doctors do and can, become completely under the influence of some alcoholic in one short hour. Shall we excuse them, and say they are not responsible and call it a disease? If this is a disease how easy the cure compared with all other forms of sickness? Take a man who has drunk continually for ten years; all at once he stops and never drinks again. We know them by the dozens. What has done it? What has cured the man of this "disease?"—this malady which has beggared his family for years and ostracised him from society? Is it any drug? Is it any change in some organ, where structural lesions had existed? Not at all. *He simply made up his mind not to drink.* Henceforth everything progresses well; the man is in perfect health; his business is excellent—never better; his family is happy; he conducts large and profitable enterprises; his mind was never as clear—his reasoning powers never so acute. This man, then, has been cured of his so-called desire without any medication, *by a simple process of his will*, and he remains so to the end of life. No other disease, the history of which I am acquainted, is cured like this. Some may urge, however, that this is an exceptional case; that it is a recent case; that the appetite had not obtained control of the man. To this I say, that a close analysis of more than 2,500 cases convince me—that a man who has drank ten, twenty or thirty years stops just as easy as one who has been addicted to alcoholic excess for a single month.

At this stage in the discussion I shall be met with the assertion, that since I maintain that it is not a disease, and inasmuch as I say that it is all nonsense to talk about the irresponsibility of men in regard to drinking liquor, some will begin to talk about the sins of the fathers being visited upon the children, even to the third and fourth generation—and the anxious and kind-hearted relative and parent commences to excuse the drunkard on account of inheritance. They refer you to the scheme inaugurated by Laban for changing the color of his cattle, sheep and goats, and ask if you deny the fact that paternal impressions aud peculiarities are not at the present day transmitted to offspring.

I do not propose to discuss the matter of heredity. It is a topic itself, the consideration of which would consume more space than I nave at my command, but I do desire to state a few facts. Of 541 men admitted to the Washingtonian Home during 1880, only eight (8) dared to say that they thought the appetite which they persist in appeasing was the result of hereditary influence: and of 1,525 admitted during the past four years, *only thirty-one* claim any influence of this kind. One other fact I desire to state and then I pass to another point. This is a matter of experience; a matter of observation. A man is reared in a moral or religious family, where liquor is never seen, and where the influence of heredity can by no possible way exercise any influence, but where indulgence and lack of early discipline is prevalent, and a general easy and indolent time is permitted. Let such a young man acquire the habit of drinking, and his reformation is more difficult than that of a young man coming up in a family where liquor is freely used, but very early he is taught to care for himself, to work, to obey those in authority, and to assume some of the responsibilities of life. That is to say, idleness and want of government are more important factors as causes of drunkenness than heredity.

I regard the testimony of the Bible of considerable importance on this subject. Diseases of various kinds are described to some extent, and vice and wickedness pretty sharply denounced. At no place, as far as I am aware, is a "woe" pronounced on a disease, or its victims precluded from the enjoyment of Paradise. Not so, however, with whisky drinking. The word of God, particularly the New Testament, speaks out in no uncertain manner in regard to the viciousness and sinfulness of drunkenness:

I. "A *woe* on drunkard-makers:" Hab. ii, 15.

II. "A *woe* on the wine-drinker:" Isaiah xxviii, 1, et seq. Prov. xxiii, 29, 30.

III. Drunkenness condemned as other sins: Luke xxi, 34; Rom. xiii, 13, 14.

IV. Drunkenness excludes from Heaven: Gal. v, 19, 20, 21: 1 Cor. vi, 9, 10.

THE TREATMENT OF INEBRIATES.

Having now discovered the first causes of inebriety, and found that a want of education among the youth of our country is the primal element which produces a habit which, in after years, is thrown off with the greatest difficulty; that the habit is formed so insiduously that those mostly concerned are hardly aware of its power before the victim is within its grasp, and *secondly:* having demonstrated by actual observation and by statistics, that after the habit is formed it is nearly impossible for men to entirely overcome the appetite—either from the fact that they cannot be aroused to the importance of accepting all the safeguards which are accessible to all, or because from defects in their early discipline they yield to drink as a panacea for all trouble, and finally, experience having convinced nearly every one having much to do with this unfortunate class, that many do not seem to care for any reform; do not care to stop a habit which not only brings sorrow and degradation to the household, but burdens on the State—what then shall be the treatment?

It seems to me that our labors should be fairly systematized among three classes:

1st. Among the young, who know nothing of the habit.

2d. Among those who seem honestly to reform, and are willing to accept the means which will produce it.

3d. Among the uncontrollable, incorrigible class, who profess to desire a reformation, but, after short periods of abstinence, repeatedly fall, and have not only ruined themselves but have discouraged and rendered miserable their relatives and acquaintances. This class is really a burden to the community.

As a result of my investigations, I am particularly impressed with the idea that the largest amount of temperance work should be among the young; that by every means possible they should be prevented from forming the habit of *moderate drinking;* that the law against selling liquor to minors should be *rigid and enforced.*

The youth of our country, of all nationalities, should be made to feel that the cultivation of the taste for liquor is a dangerous habit; that alcohol in all its forms is a drug to be dreaded and avoided as strychnine or aconite. Let parents in all conditions of life be made to feel, and educated to believe, that it is a dangerous practice to allow, and a fearful responsibility to encourage their children to drink the mild wines, beer or cider.

While I am writing this section of my paper a gentleman fifty-two years of age, and one who has experienced all the degradation possible from drink; has lost his fortune; ruined his health, and became separated from his family, said to me: "Forty years ago I attended church three times each Sunday, and listened to sermons one hour in length each time, and in addition attended Sabbath school, and during all these religious exercises for years *I never heard a word in regard* to temperance. I commenced gradually and knew nothing of its dangers." This is true of hundreds and thousands. Such a scheme as I have mentioned is, I am happy to say, already being inaugurated and has been pushed forward by some church organizations with more than usual success: but it is not general enough; it reaches only a comparatively small number of those who should be benefited by such efforts.

What shall be done with the class who have already acquired the habit?

Probably about twenty per cent. can be reformed the first time their attention can be arrested long enough to keep them perfectly sober. This can be done by Homes, Asylums, Societies, Prayer Meetings, Ribbon Clubs, etc. Any way by which the whisky can be eliminated from the system, and a few days rest for the body be taken, and then followed by encouraging words; a definite plan of work, and a resolution never to associate with former companions. If the question is asked, Why have homes and asylums for inebriates been established and maintained at a cost of money and labor, if the habit of inebriation is not a disease, and only twenty per cent. saved ? I would reply, that after temperance organizations, and societies, and churches, and clubs have done all that is possible, some of the most promising of those reformed will relapse from causes which I have just enumerated.

These men need a place where they can acquire strength ; where, separated from their companions, their *moral sense* can be cultivated and strengthened. Some men reform in a moment ; with others it takes months, perhaps years. Many men use alcoholics for years without the impairment of any function of the body ; others are injured in nearly every organ. For those who need considerable time to perfect a reformation, and that class, too, whose constitutions have been injured by the voluntary use of alcoholics, and who, from habit and association, have not the moral force to say No to an invitation to drink, to such, homes and asylums are houses of refuge. Reformation is a matter of development—not a "presto-change" affair—not done in the twinkling of an eye—but a matter of growth, a matter of development. A man who has been addicted to the inordinate use of whisky is never safe. He must be on his guard. A reformed man must be taught, and the lesson must be well learned, that let come what will—sickness, trouble, death, reverses—singly or all combined—that he cannot, with any safety, touch a drop, or he will fall.

Men who have stopped drinking, but are not guided by a high motive ; those who have not made up their minds that for them it is wicked to use alcoholics because of an acquired taste, will constantly be making experiments. They see the great majority of men drinking a little every day, and they believe that they can do the same. They make up their minds to drink only beer after supper, or mineral water in the forenoon, some cinchona in the afternoon and beer in the evening. They proceed to experiment, and in about 999 cases in a thousand they fall. These men, then, are never safe ; their burden is to fight an appetite which was formed before they knew its danger. Asylums for the reception of this second class should multiply, they must be increased, and the work in this generation will never cease. If it becomes less it will be due to the fact that the influence of innumerable temperance educators is being felt by the youth of this country. Until this is done we must provide for the victim of intemperance.

What is to become of the remaining eighty per cent.? I stated, it must be remembered, that I thought about twenty in each hundred could be quickly reformed, if we could get access to them. What about the remaining 80?

Quite a number will reform after repeated trials and failures; a few, after education upon the subject, will drink less—are improved, but not reformed. But a considerable number go to form the third class, the description and treatment of which I will now speak.

These cannot be reformed, either through the influence of temperance organizations, or by the moral strength acquired in a reformative institution. These are the uncontrollable, incorrigible, disquieted men, who have not only discouraged but rendered miserable their relatives, and all their acquaintances. They have never learned to obey; they are undisciplined, and generally lack all feeling of responsibility. They are the men who beat their wives and starve their children; the men who steal the hard-earned money from their wives' purse, and the knives and napkins from their tables (as has happened within my own observation within the past two weeks) to buy rum, and, when perfectly sober, you expostulate with them, they will laugh, and assure you that they know their own business. The State should assume the guardianship of this class, and should put them in an institution whose management should be remarkable for its kind administration of affairs, and for a discipline most rigid. This institution should be situated on a farm, and men of this class sent there for not less than two years. From ten to twelve hours work every day during this time of commitment, combined with judicious and strict discipline, with the assurance that the State would again assume charge of them if they returned to their former habits, would produce an effect on these men, which would result in the reformation of nearly all. Let them learn that it is *some one's business* if they become absolutely indifferent to all of the responsibilities of life. In the language of Dr. Bucknill: "These men are not to be 'coddled' in luxurious indolence, nor impressed with the pernicious idea that they are interesting but helpless objects of social and psychological science."

